# Effect of vacuum plasma treatment duration on physicochemical, mechanical, and biocompatibility properties of bacteriophage-incorporated PVA/duck egg white nanofibers

**DOI:** 10.1039/d6ra01532h

**Published:** 2026-07-02

**Authors:** Kaushik Kokil Nath, Dhirangkana Bora, Orison Waikhom, Akuleti Saikumar, Subrata Mishra, Bikash K. Das, Bibhusita Baishya, Biplob Mondal, Laxmikant S. Badwaik, Nirmal Mazumder, Manabendra Mandal, Suman Dasgupta, Rajib Biswas, Gazi Ameen Ahmed

**Affiliations:** a Laboratory for Plasma Processing of Materials, Department of Physics, Tezpur University Tezpur Assam 784028 India; b Applied Microbiology and Biotechnology Laboratory, Department of Molecular Biology and Biotechnology, Tezpur University Tezpur India; c Sensors and System Engineering Laboratory, Department of Electronics and Communication Engineering, Tezpur University Tezpur India; d Department of Food Engineering and Technology, Tezpur University Tezpur India; e Metabolic Disease Biology Laboratory, Department of Molecular Biology and Biotechnology, Tezpur University Tezpur India; f Nanoscience and Soft-Matter Laboratory, Department of Physics, Tezpur University India; g Department of Biophysics, Manipal School of Life Sciences, Manipal Academy of Higher Education Manipal India nirmal.mazumder@manipal.edu

## Abstract

Chronic wound infections caused by multidrug-resistant (MDR) *Pseudomonas aeruginosa*, pose a critical challenge in the clinical area, particularly for burn victims and immunocompromised patients where conventional antibiotic therapies continue to fail. This study reports the development of vacuum oxygen (O_2_) plasma treated electrospun nanofibers composed of polyvinyl alcohol (PVA), duck egg white (DEW), and *P. aeruginosa*-specific bacteriophage vB_PaP_DMTU_1 (10^9^ PFU mL^−1^). By varying plasma exposure (1, 3, 5, and 10 min) at a fixed discharge power of 144 W (0.6 A), we precisely tuned surface chemistry to enhance wound dressing performance. Results demonstrate that short exposure durations (1–3 min) introduce oxygen-containing polar groups, significantly improving crystallinity (up to 46.7%), wettability (water contact angle from 99° to 49°), and total surface energy. These shifts resulted in a 72% increase in tensile strength, with a water vapor transmission rate (1981–2074 g m^−2^ 24 h^−1^) within the ideal clinical range for moist environment for wound healing. Crucially, these optimized conditions preserved phage viability achieving potent disruption of biofilm with large inhibition zones (41–50 mm) against MDR *P. aeruginosa*. In contrast, prolonged exposure (≥5 min) led to inactivation of phages, *via* capsid disruption. The nanofibers demonstrated superior biocompatibility, with minimal haemolytic activity (<0.9%) and high cell viability (>96% at 24 h). This study demonstrates that controlled vacuum O_2_ plasma treatment for 1–3 minutes produces mechanically robust, phage-active, and biocompatible PVA/DEW nanofibers offering an antibiotic-free strategy for combating *P. aeruginosa* in chronic and burn wound infections.

## Abstract 2

Chronic wound infections caused by multidrug-resistant *Pseudomonas aeruginosa*, pose a critical challenge, particularly for burn victims and immunocompromised patients where conventional antibiotic therapies continue to fail.

## Graphical abstract

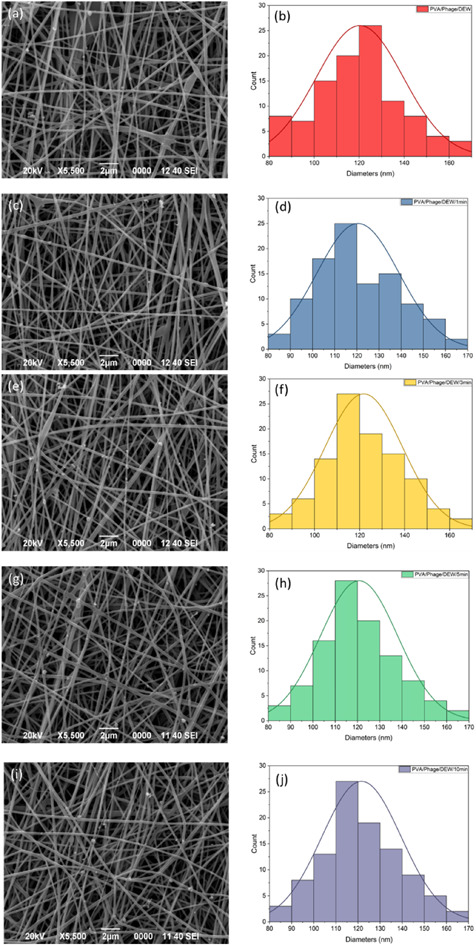

## 1 Introduction

Chronic wounds and infections with bacterial resistance pose a significant challenge to the healthcare system, resulting in delayed healing and increased morbidity. The rise of multidrug-resistant (MDR) pathogens such as *Pseudomonas aeruginosa* has reduced the efficacy of conventional antibiotic therapies, prompting the exploration of alternative antimicrobial strategies.^1,2^ This Gram-negative bacterium exploits the disrupted tissue environment through a complex virulence mechanism, establishing persistent biofilm infections that can resist antimicrobial treatment.^3,4^ Bacteriophages are viruses that selectively infect and destroy bacteria, and have emerged as promising biocontrol agents due to their specificity and ability to target antibiotic-resistant bacterial strains.^5,6^ Among innovative drug delivery platforms, electrospun nanofibers have gained considerable attention for wound dressing applications owing to their high surface area, excellent porosity, and controlled release of active therapeutic agents.^6^ These dressings have promoted healing of acute and chronic wounds through multiple mechanisms such as protecting against infection, delivering localized therapeutics, and enabling tissue regeneration using biocompatible synthetic and natural polymers.^5,7^ Optimal wound dressings maintain moist environment, promote tissue regeneration and minimize injury while ensuring patient comfort. Integrating bacteriophages into biocompatible nanofiber matrices offers distinct advantages over traditional wound treatments. This approach not only protects phage viability but also enables sustained, localized antimicrobial action directly at the wound site. Phage loaded nanofiber dressings surpass traditional bioactive formulations incorporating active pharmaceutical ingredient (APIs) like antibiotics, anti-inflammatories, vitamins, and growth factors.^7^ Preclinical models demonstrate that this combination has demonstrated effectively reduces bacterial load, preventing biofilm formation, and accelerating tissue regeneration. Therefore, approach with phage-loaded nanofiber wound dressings can addresses both infection control and wound healing, highlighting their potential for future clinical translation.^4,7^

Cold plasma treatment has recently gained considerable attention for surface modification of polymer biomaterials, due to its ability to tailor the surface properties for biomedical applications.^8,9^ Plasma, a partially ionized gas composed of electrons, ions, excited gas molecules, atoms, free radicals and photons, provides an environmentally friendly alternative to surface modification methods. Unlike wet chemical process, plasma treatment modifies polymer surface properties and chemistry without affecting bulk characteristics, offering faster processing with shorter treatment times.^10^ While atmospheric-pressure plasma systems can operate without vacuum infrastructure and are widely used for surface modification, they can exhibit spatial non-uniformities in discharge at larger scales. In contrast, vacuum plasma systems operate at reduced pressure (in this study: −100.3 kPa) and provide precise, spatially homogeneous, and highly controlled surface modification conditions, making them particularly advantageous for delicate, phage-loaded biomaterials where treatment uniformity and reproducibility are critical.^11^ Plasma treatment introduces functional groups to the polymer surface, significantly improving hydrophilicity and biocompatibility by increasing the surface energy and hydrophilicity with the introduction of polar groups (*e.g.*, hydroxyl and carboxyl). Plasma treatment facilitates superior cell attachment and proliferation while ensuring immediate interaction between the antimicrobial surface and the wound exudate, a critical factor for effective bacterial inhibition.^10,12^

Duck egg white (DEW) is a protein-rich biomaterial that comprises of 10.7% protein, with essential amino acids, like methionine that support tissue regeneration.^13^ Raw DEW, exhibits remarkable adhesiveness, high water holding capacity, and contains antimicrobial proteins, including lysozyme and ovotransferrin, making them a promising candidate for advanced wound dressings.^14,15^ However, DEW alone lacks the properties necessary for stable fibre formation during electrospinning. Polyvinyl alcohol (PVA) is a synthetic biopolymer, known for its biocompatibility, water solubility, non-toxicity, and superior fiber forming capabilities.^16^ While PVA provides a stable mechanical scaffold, its biological activity is limited, therefore blending DEW with PVA creates a composite that facilitates electrospinning of robust nanofiber matrices, where DEW functions as bioactive components. This combination yields mechanically stable, moisture retaining and biologically active dressing capable of accelerating wound repair.

Our work, is build up upon the work of previous research done by Ojah *et al.*^17^ and the current study investigates the effect of oxygen (O_2_) plasma treatment duration on electrospun nanofibers composed of polyvinyl alcohol (PVA), bacteriophage (Phage), and duck egg whites (DEW). This bacteriophage incorporated electrospun nanofiber mats provide an innovative antimicrobial wound dressing alternative. By combining the high surface area and extracellular matrix (ECM) mimicking structure of nanofibers with the bacteriophage, these nanofibers offer sustainable bacterial removal while enhancing cellular interactions and wound healing processes. The results showed an optimized treatment time preserves bacteriophage functionality while preventing growth of *P. aeruginosa* at the wound sites. This study aligns with green electrospinning principle, utilizing ecofriendly, non-toxic polymers and solvent for biomedical application.^18^ To evaluate the impact of plasma treatment, electrospun PVA/Phage/DEW nanofibers were exposed to O_2_ plasma for durations of 1, 3, 5 and 10 minutes, while keeping constant operating parameters including electrode gap (55 mm), applied power (144 W) and gas flow rate (20 mL min^−1^). Physicochemical properties of untreated and plasma-treated nanofibers were characterized using multiple techniques scanning electron microscopy (SEM), attenuated total reflectance Fourier-transform infrared spectroscopy (ATR-FTIR), powder X-ray diffraction (P-XRD), texture analyser, and contact angle measurements. Additionally, water vapour transmission rate was also studied to evaluate their potential for wound healing applications. *In vitro* biocompatibility was assessed through haemolytic activity assay, antithrombogenic tests, and methylthiazolyldiphenyl-tetrazolium bromide (MTS) assays. By subjecting both untreated and plasma treated nanofibers to various characterization techniques, and corelating observed properties with plasma treatment duration, determined optimized conditions for maximizing therapeutic potential while maintaining material integrity and biological safety.

## 2 Materials and methods

### 2.1 Materials

Duck eggs were sourced from a local poultry farm near Tezpur University (Napaam, Tezpur, India). Polyvinyl alcohol (PVA) (fully hydrolysed, *M*_w_ 84–125 kDa) was procured from Merck (India). All chemicals were used as received without any further purification. Ultrapure distilled water was obtained using a Millipore Milli-Q system (USA).

### 2.2 Methods

#### 2.2.1 Preparation of PVA/DEW precursor solutions

Freshly laid duck eggs were collected, and the egg white (EW) was carefully separated from the yolk. The EW was homogenized at 1000 rpm to ensure uniformity and then filtered through a 100 µm pore-size filter to remove foam and impurities. For preparation of 8% (w/v) polyvinyl alcohol (PVA) aqueous solution, PVA powder was dissolved in deionized water and stirred continuously at 80 °C for 4 hours. The resulting solution was allowed to stand at room temperature overnight to eliminate residual air bubbles, ensuring suitability for subsequent electrospinning. Subsequently, the egg white (duck) and PVA solutions were combined in a predetermined ratio of 2 : 1 (PVA : DEW) (provided in SI Fig. S1) with gentle stirring for 60 minutes to ensure the mixture is mixed homogenously.

#### 2.2.2 Preparation of bacteriophage

Bacteriophage vB_PaP_DMTU_1 was isolated from sewage water which exhibit significant biocidal and antibiofilm activity against *Pseudomonas aeruginosa*. The morphological and genomic characterization, biocidal and antibiofilm potential of the phage were previously detailed by Bora *et al.*, 2026.^19^ Bacteriophages were allowed to replicate with its specific host strain (enrichment step) in sodium–potassium buffer for 12 hours at 37 °C and 120 rpm. Phage lysate was treated with 1% v/v chloroform and centrifuged at 10 000 rpm for 20 minutes and then filtered through 0.22 µm syringe filter to remove any bacterial contaminants. The lysate was concentrated by centrifuging in a spin filter of pore size 10KD (Amicon Ultra-15, Merck Millipore, and Ireland). The concentrated phage solution is titred using double-layer agar plating method and final concentration is found to be 10^9^ PFU mL^−1^ and stored at 4 °C.

#### 2.2.3 Electrospinning of PVA/Phage/DEW solutions

Bacteriophage was incorporated into a PVA/DEW solution at a concentration of 10^9^ PFU mL^−1^. The solution was stirred gently for 45 minutes to ensure uniformity and allowed to settle down before electrospinning. The solutions were loaded into a syringe fitted with 20G needle and electrospun at a high voltage of 22 kV with a flow rate of 0.8 mL h^−1^. A stainless-steel drum collector, covered with aluminium foil measuring 130 mm × 130 mm serves as the grounded static collector. Nanofibers are deposited onto the aluminium foil at a tip-to-collector distance of 130 mm under ambient conditions (temperature: 27 °C and relative humidity: 53%). The resulting nanofiber mats were stored in a vacuum desiccator for further characterization.

#### 2.2.4 Vacuum plasma treatment

The vacuum plasma system manufacture by Eltech Engineers Pvt. Ltd was utilized for the plasma treatment process. The system consists of two plates: a bottom plate measuring 170 mm × 140 mm and the top plate measuring 150 mm × 110 mm, with a fixed separation of 55 mm. Prior to plasma treatment, the nanofibers were carefully peeled from aluminium foil and cut into pieces of required size. These pieces were then positioned at the bottom plate of the vacuum plasma system. O_2_ plasma treatment was applied with a constant power of 144 W (0.6 A) at a chamber pressure of −100.3 kPa, with treatment durations of 1, 3, 5 and 10 minutes. Throughout the process, oxygen O_2_ gas was supplied at a steady flow rate of 20 mL min^−1^, controlled by a mass flow controller integrated into the system. Under these discharge conditions, the vacuum plasma remained stable and homogeneous, effectively covering the entire surface of the plate and distributing uniformly within the gap of two plates. It is important to note that this experimental design differs from our previously reported study,^20^ in which the same vacuum plasma system was employed but treatment duration was fixed at 60 s while power was varied across 96, 144, 192, and 240 W. The present study complements that work by separating treatment duration as the independent variable, providing insight into how longer plasma exposure, rather than higher power, affects the physicochemical and biological characteristics of the nanofibers (Table 1).

**Table 1:** Visual observation of the discharge glow between the electrodes during the vacuum O_2_ plasma treatment of nanofiber mats at varying time intervals

Discharge glow between the electrodes	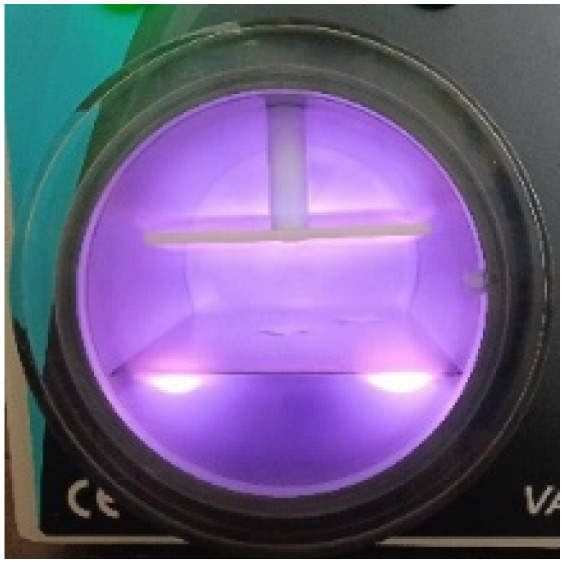	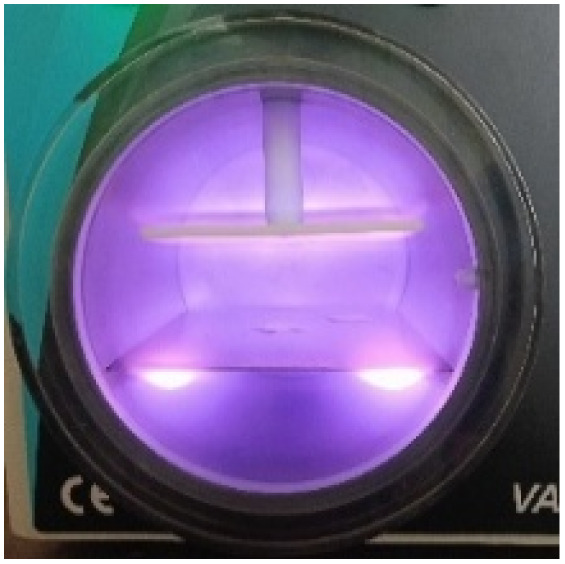	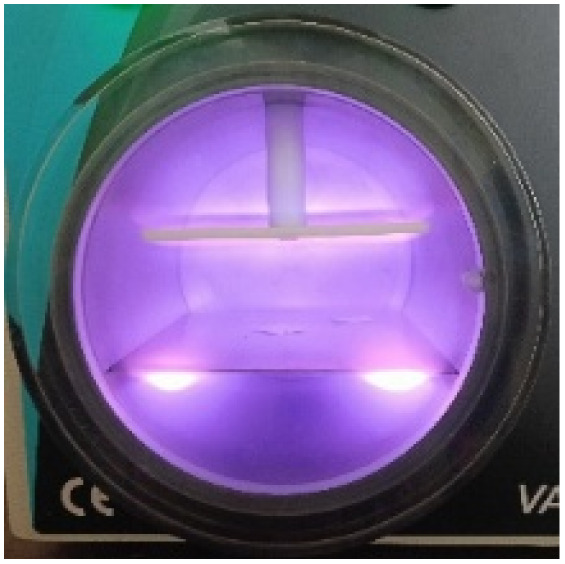	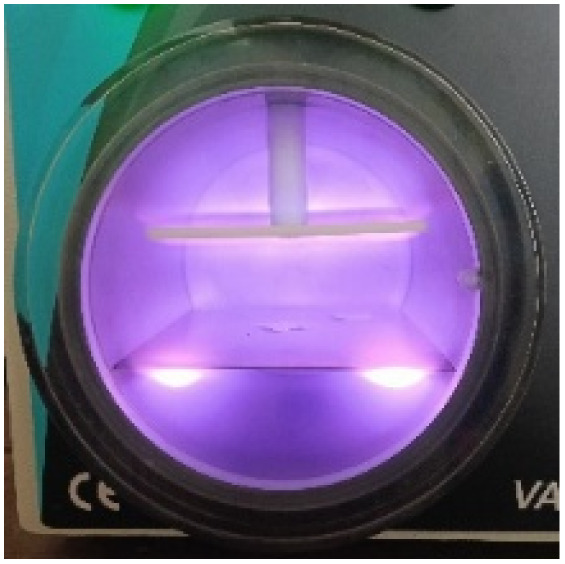
Plasma treatment time	1 min	3 min	5 min	10 min

## 3 Characterization techniques

### 3.1 Surface morphology

The surface morphology of the synthesized nanofibers (PVA/Phage/DEW) were analysed using scanning electron microscope (SEM, JEOL JSM-7200F, Tokyo, Japan). For comparative analysis, SEM imaging of O_2_ plasma-treated nanofibers was performed for different time intervals. For the measurement process, nanofibers along with the aluminium foil were cut into 5 mm × 5 mm pieces and coated with a thin layer of gold using a low vacuum auto fine coater (JFC-1600, Tokyo, Japan). ImageJ software was applied for calculating the mean of nanofiber diameter by measuring 100 randomly selected fibres (*n* = 100).

Energy-dispersive X-ray spectroscopy (EDX) was performed using an INCAx-Sight microanalysis system (Oxford Instruments, Model 7582), coupled with the SEM, for elemental analysis. The system utilized a silicon (lithium-drifted) crystal detector of the EDX with an acquisition rate of 50 000 cps. The detector's Super Atmosphere Supporting Thin Window (SATW) ensured high resolution at low energies, achieving resolution of 137 eV at 5.9 keV, with a minimum quantification limit of 0.01 wt%.

### 3.2 Chemical analysis

For identifying the chemical structure and composition of both untreated and plasma treated nanofiber PVA/Phage/DEW mats were analysed using attenuated total reflectance Fourier transform infrared (ATR-FTIR) spectroscopy (IMPACT 410, NICOLET, USA). Spectra were recorded from a 200 µm diameter sampling area, averaging 200 scans and a spectral resolution of 4 cm^−1^. Data collection was performed in transmittance mode over a spectral range of 4000–600 cm^−1^.

### 3.3 P-XRD analysis

X-ray diffraction (XRD) patterns of the samples were recorded over a 2*θ* range of 10° to 40° using a powder XRD instrument (D8 Focus, Bruker, Germany). The crystallinity percentage of both treated and untreated PVA/Phage/DEW nanofiber mats were calculated from the XRD pattern by differentiating the crystalline and amorphous regions. This degree of crystallinity was determined using following equation:1
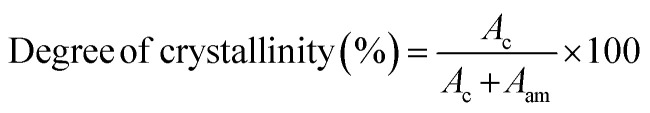
Here, *A*_c_ represents the area under crystalline peak, while *A*_am_ denotes area under the amorphous regions.^21^

### 3.4 Mechanical studies

The mechanical properties of the fabricated nanofiber mats were evaluated using a texture analyser (TA-XT plus, Stable Micro System, UK) equipped with a 5 kg load cell, in accordance with the ASTM D882-02 standard. As per ASTM D882-02, tensile strength is calculated as force per unit cross-sectional area (width × thickness) rather than per unit mass, as this standard is specifically designed for thin plastic sheeting and polymer films. This approach is therefore appropriate for electrospun nanofiber mats and is consistent with established literature for PVA-based systems. Samples, sized at 60 mm × 10 mm, with an effective gauge length of 40 mm between the grips, were tested with a grip separation of 50 mm and a test speed of 2 mm s^−1^.^22,23^ To ensure sample uniformity, all samples (*n* = 5 specimens per group) were cut from the central area of the electrospun mat where deposition was more consistent. Tensile strength and elongation at break values are reported as mean ± SD (*n* = 5). The thickness of each specimen was measured at five random points using a digital micrometer. The tensile strength (TS), and elongation at break (EAB) of the mats were calculated using the provided formulas:2
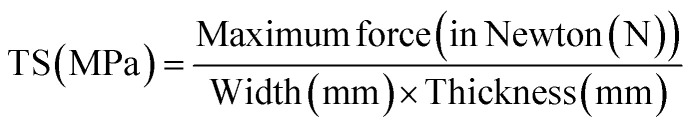
3
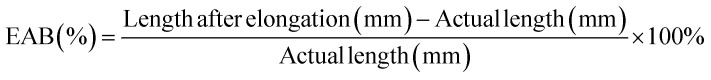


The tensile strength and elongation at break (%) were reported as mean ± standard deviation (SD).

### 3.5 Wettability measurement

The static contact angles of PVA/Phage/DEW nanofiber mats, both untreated and plasma treated were determined using a contact angle meter (DMs-401, Kyowa Interface Science®, Japan) employing the sessile drop method. The instrument is equipped with an automated syringe–liquid delivery system, a CCD camera operating at 60 fps, which enabled capture of the initial (*t* = 0 s) and a white light source. The auto captured images were analysed using FAMAS® software integrated with the instrument. Both polar (deionized water) and non-polar (ethylene glycol) probe liquids were used for the measurements. Nanofiber mats were cut into 10 mm × 10 mm sections and carefully mounted onto microscopic glass slides to ensure that the native surface remained flat and undisturbed during the measurement process. A droplet volume of 2 µL was dispensed for each measurement, and data were collected at five independent locations on each sample. All measurements were conducted at ambient temperature under normal laboratory humidity conditions. The surface energy (*γ*_s_) of the nanofibers was determined using the Owens–Wendt–Rabel–Kaelble (OWRK) method.^24^ In this approach, droplets of double-distilled water (polar liquid) and ethylene glycol (non-polar liquid) were deposited on the nanofiber surface, and both images and contact angles were recorded immediately (within 1–2 seconds) upon contact to minimize the effects of droplet absorption, swelling or evaporation. The total surface energy of the PVA/Phage/DEW and PVA/Phage/DEW/O_2_ nanofibers was calculated according to the following relation:

4 *γ*_s_ = *γ*^p^_s_ + *γ*^d^_s_,

where *γ*^p^_s_ and *γ*^d^_s_ are the polar and dispersive components of the surface energy (*γ*_s_), respectively. To quantify *γ*_s_, the polar and dispersive components were derived by solving the system of equations obtained from contact angle measurements with water and ethylene glycol:

5 (*γ*^d^_s_)^0.5^ + 1.53(*γ*^p^_s_)^0.5^ = 7.80(1 + cos *θ*_W_),

6 (*γ*^d^_s_)^0.5^ + 0.76(*γ*^p^_s_)^0.5^ = 4.80(1 + cos *θ*_E_),

where *θ*_W_ and *θ*_E_ denote the contact angles of water and ethylene glycol, respectively. Measurements were performed at multiple positions across a 10 mm × 10 mm sample area to ensure representative data.

### 3.6 Water vapour transmission rate (WVTR)

The water vapour transmission rate (WVTR) of the nanofiber mats was evaluated using a gravimetric method adapted from established protocols.^25,26^ Both untreated and plasma treated mats PVA/Phage/DEW treated for 1, 3, 5 and10 minutes were attached onto glass bottles containing 4 g of anhydrous CaCl_2_ (0% relative humidity, RH) and sealed tightly along the edges using laboratory Parafilm. The initial weight of each bottle, including the attached nanofibers was measured using a digital balance (Mettler Toledo, ME204) before placing them inside a desiccator containing a saturated NaCl solution (75% RH). The weight changes recorded daily at 24 hours intervals over 10 days. The WVTR was calculated by dividing the slope of the weight gain (in grams) *versus* time (in days) by the product of the mat's exposed surface area (in m^2^) and saturation vapour pressure (2.37675 kPa at 25 °C and 75% RH). Measurements were conducted in triplicates (*n* = 3), and the average values are reported.7
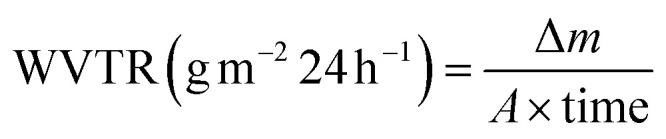
where Δ*m* is the mass of water vapour transmitted (g), *A* is the exposed surface area of the nanofiber mat (m^2^), and time is the measurement duration (24 h).

### 3.7 Antibacterial study

Antibacterial activity of untreated and plasma-treated PVA/Phage/DEW nanofibers was evaluated against *Pseudomonas aeruginosa* using agar disk diffusion method. This method was specifically chosen to assess the diffusion driven release of bacteriophages from the nanofiber mats, which simulates the delivery of bioactive agents into the wound sites. Log phased *P. aeruginosa* strain (MTCC2297) was spread uniformly in Muller–Hinton Agar (MHA) plate and circular nanofiber samples of 20 mm diameter were placed on the agar plate, incubated at 37 °C for 24 hours, and inhibition zones measured to assess antibacterial efficacy. All assays were performed in triplicate (*n* = 3) and results are expressed as mean ± SD.

### 3.8 Haemolytic activity test

Fresh goat blood was procured from a local butcher shop (Near Tezpur University, Napaam, Tezpur, Sonitpur, Assam) in a heparinised tube for haemolysis assay. Plasma-treated and untreated PVA/Phage/DEW nanofiber mats each of size 40 mm × 40 mm, were incubated with 150 µL of goat blood containing a 10% red blood cell (RBC) suspension diluted with 0.9% NaCl. The final volume was 2 mL with 0.9% NaCl, and the samples were incubated at 37 °C for 1 hour. A negative control consisted of blood mixed with 0.9% NaCl, and a positive control used blood mixed with distilled water (without the mats). After incubation, the samples were centrifuged at 10 000 rpm for 10 minutes. The supernatant was analysed for free haemoglobin content using a UV-visible spectrophotometer at a wavelength of540 nm. The haemolysis percentage was calculated using the following formulae,8
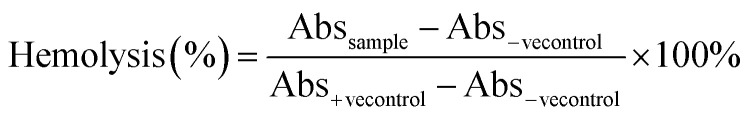
where Abs_sample_ is the absorbance of the test sample at 540 nm, Abs_−vecontrol_ is the absorbance of blood in 0.9% NaCl (no lysis), and Abs_+vecontrol_ is the absorbance of blood in distilled water (100% lysis).

### 3.9 Antithrombogenic

The antithrombogenic properties of O_2_ plasma treated and non-treated PVA/Phage/DEW nanofibers were evaluated with goat blood. 1 mL of anticoagulant citrate dextrose (ACD) blood was incubated with plasma treated and non-treated PVA/Phage/DEW nanofibers (20 mm × 20 mm) separately at 37 °C for 1 h. Then, 0.2 mL of blood was withdrawn from each sample and placed on a sterilized glass plate. Clotting was initiated by adding 0.02 mL 0.1 M CaCl_2_ to the citrated blood. After 15 and 60 minutes intervals, the resulting blood clot on each glass plate were scrapped, fixed in 2 mL of 36% (v/v) formaldehyde solution for 10 minutes, washed with distilled water, dried and weighed. The ACD blood, without nanofiber incubation, served as the control.

### 3.10 Cell viability assay

Mouse RAW264.7 macrophage cells were sourced from National Centre for Cell Science (NCCS), Pune, India, and were maintained in Dulbecco's Modified Eagle Medium (DMEM) supplemented with 10% Fetal Bovine Serum (FBS) and 1% penicillin–streptomycin solution. The cells were cultured at 37 °C in a humidified incubator with 5% CO_2_, following standard protocols. For the cytotoxicity assay, RAW264.7 macrophages were seeded at a density of 1 × 10^4^ cells per well in a 96-well plate and incubated for 24 hours to allow adherence. Subsequently, the cells were treated with test samples at a concentration of 1 mg per well for varying durations (6, 12, and 24 hours). Post-treatment, 0.5 µg µL^−1^ MTT reagent was added to each well, and the plates were incubated for an additional 4 hours at 37 °C to allow formazan crystal formation. The formazan crystals were then dissolved in 100 µL of acidic isopropanol, followed by 30 minutes incubation at 37 °C. Cell viability was assessed by measuring absorbance at 570 nm using a Multiskan GO Microplate Spectrophotometer (Thermo Scientific, Finland). Absorbance values were normalized by subtracting the acidic isopropanol blank. The absorbance of untreated cells (exposed to medium only) was set as 100% cell viability (control). The percentage of cell viability was calculated using the following formula:9
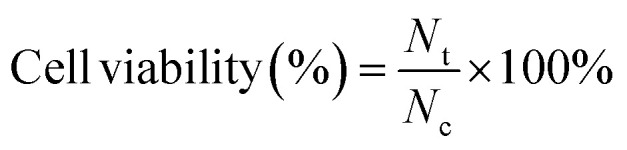
where *N*_t_ is the absorbance at 570 nm of treated cells, and *N*_c_ is the absorbance at 570 nm of untreated control cells.

### 3.11 Statistical analysis

All experiments were conducted in triplicates (*n* = 3), and the results are expressed as the mean ± standard deviation (SD). The statistical significance of differences among the tested samples was determined using one-way analysis of variance (ANOVA) followed by Tukey test (Origin Software). A *p* value < 0.05 is considered statistically significant for hemocompatibility and MTT assay studies.

## 4 Results and discussion

### 4.1 Surface morphological analyses

**Fig. 1:**
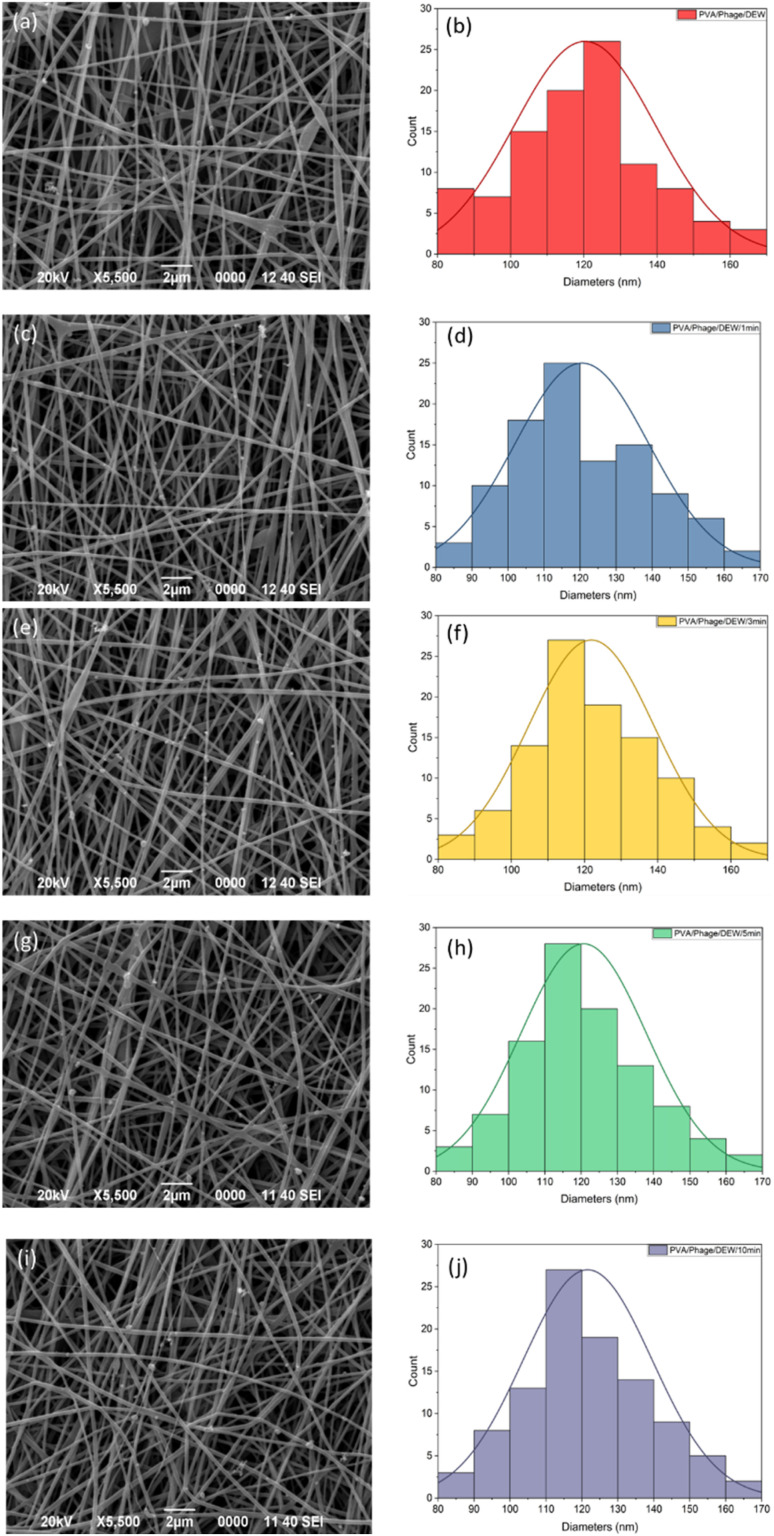
SEM images (2 µm scale bar) and fiber diameter distribution histograms of electrospun nanofiber mats: (a and b) untreated PVA/Phage/DEW nanofiber mat (PVA : DEW ratio of 2 : 1); (c and d) plasma-treated PVA/Phage/DEW nanofiber mat (PVA : DEW ratio of 2 : 1) for 1 min; (e and f) for 3 min; (g and h) for 5 min; (i and j) for 10 min; all images acquired at 5500× magnification.

The morphological analysis using SEM imaging (Fig. 1) confirmed that the oxygen (O_2_) vacuum plasma treatment had a negligible effect on the bulk properties of PVA/Phage/DEW nanofiber. All nanofibers maintained a nanoscale, randomly oriented, and beads free structure irrespective of the treatment duration. As shown in Table 2, the average fiber diameters remained consistent for both treated and untreated nanofibers. The untreated mats (0 min) exhibited a diameter of 121.81 ± 1.70 nm, while diameters after plasma treatment for 1, 3, 5, and 10 minutes were 121.08 ± 1.18 nm, 121.24 ± 1.43 nm, 121.01 ± 1.19 nm, and 121.67 ± 1.72 nm, respectively. These results indicate that while plasma treatment successfully modifies the surface chemistry and mechanical properties of the scaffolds, it does not induce significant etching or thermal degradation for altering the overall morphology of the fibre.

**Table 2:** Average fibre diameters (mean ± SD, *n* = 100 fibres per sample) of PVA/Phage/DEW nanofibers under various plasma treatment durations

Sample	Plasma treatment time (min)	Average diameter (nm)
PVA/Phage/DEW	0	121.81 ± 1.70
PVA/Phage/DEW/1 min	1	121.08 ± 1.18
PVA/Phage/DEW/3 min	3	121.24 ± 1.43
PVA/Phage/DEW/5 min	5	121.01 ± 1.19
PVA/Phage/DEW/10 min	10	121.67 ± 1.72

For analysing elemental composition of untreated and O_2_ plasma treated nanofiber EDX was used. The data indicates a progressive increase in oxygen content from 37.76% in untreated to 40.16% after 10 minutes of plasma treatment. This confirms the successful incorporation of oxygen-containing functional groups (*e.g.* –OH, –COOH) onto the polymer surface, supporting ATR-FTIR and contact angle data. On the other hand, there is slight declination of carbon content that can be attributed to the etching of surface atoms into volatile species like CO_2_.^27^ The analysis confirms that O_2_ plasma treatment modifies the surface chemistry by progressively increasing the oxygen-to-carbon ratio.

It is acknowledged that EDX, with its bulk sampling depth has limited sensitivity for detecting thin surface-confined functional groups introduced by plasma treatment. The functional group assignments in this study are therefore based on the complementary evidence from ATR-FTIR spectroscopy, EDX elemental ratios, and contact angle data. X-ray Photoelectron Spectroscopy (XPS), which provides chemical state and bonding information from the outermost would offer definitive, quantitative confirmation of the specific functional groups (–OH, –COOH, C=O) introduced by O_2_ plasma, and is identified as a priority for future investigation (Table 3).

**Table 3:** Elemental composition of untreated and O_2_ plasma-treated PVA/Phage/DEW nanofibers determined *via* EDX analysis

Element	PVA/Phage/DEW		PVA/Phage/DEW (1 min)		PVA/Phage/DEW (3 min)		PVA/Phage/DEW (5 min)		PVA/Phage/DEW (10 min)	
	Weight (%)	Atomic (%)	Weight (%)	Atomic (%)	Weight (%)	Atomic (%)	Weight (%)	Atomic (%)	Weight (%)	Atomic (%)
C	53.17	60.80	53.73	61.10	53.42	60.78	53.33	60.59	52.12	59.36
O	43.99	37.76	44.58	38.06	45.02	38.46	45.55	38.85	46.97	40.16
Na	1.59	0.80	0.90	0.46	0.77	0.46	0.60	0.34	0.59	0.35
Cl	1.24	0.63	0.79	0.38	0.80	0.31	0.52	0.22	0.32	0.12

### 4.2 Chemical analyses

**Fig. 2:**
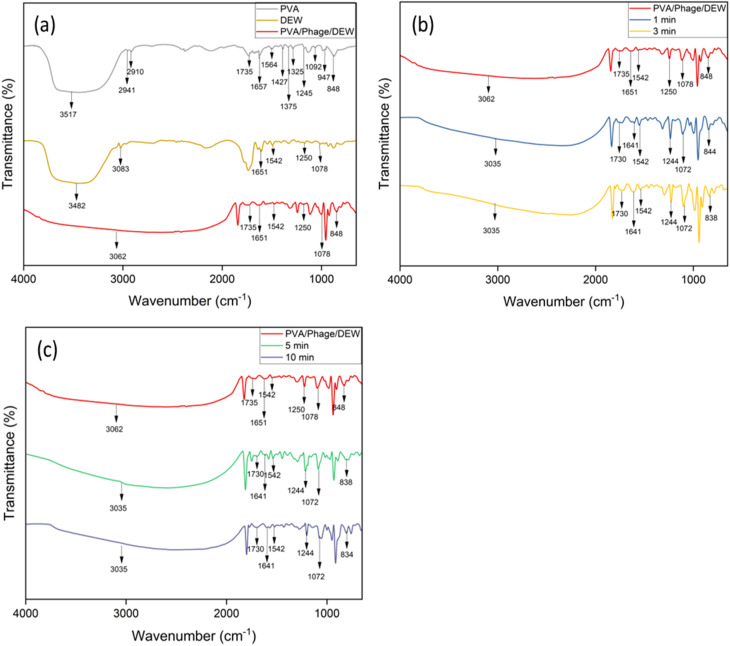
ATR-FTIR spectra of electrospun nanofibers: (a) comparative spectra of pure PVA, Duck Egg White (DEW), and untreated PVA/Phage/DEW nanofibers; (b and c) spectra of PVA/Phage/DEW nanofibers subjected to vacuum O_2_ plasma treatment for 1, 3, 5, and 10 minutes, illustrating chemical shifts due to surface oxidation.

ATR-FTIR spectroscopy was used to characterize the chemical composition of PVA, Duck Egg White (DEW), and PVA/Phage/DEW nanofibers (Fig. 2). DEW exhibited characteristic protein peaks including a broad amide A band at 3482 cm^−1^ (overlapping O–H and N–H stretching) and amide B band at 3083 cm^−1^ (asymmetric CH and NH_3_^+^ stretching). The amide I, amide II, and amide III bands appeared at 1651 cm^−1^, 1542 cm^−1^, and 1250 cm^−1^, respectively. The amide I band is attributed to C=O stretching, indicating α-helix and random coil secondary structures, while amide II and amide III arise from N–H bending coupled with C–N stretching vibrations. Additional band at 1078 cm^−1^ (C–O stretching) 457 cm^−1^ (S–S stretching) confirmed carbohydrate content and disulfide bonds.^28^ Pure PVA, displayed a broad OH stretching peak at 3517 cm^−1^ from intramolecular and intermolecular hydrogen bonding, with C–H alkyl stretching at 2941 cm^−1^ and 2910 cm^−1^. Additional peaks at 1427, 1375, 1325, 1245, and 1092 cm^−1^ represented CH_2_ bending, CH stretching, CH deformation, CH wagging, and C–O stretching, with C=C and CH_2_ stretching at 947 and 848 cm^−1^.^29^ The composite nanofiber showed a broad peak at 3303 cm^−1^ corresponding to the –OH stretching from PVA and the amide A (NH stretching) from the DEW proteins. The peak at 3062 cm^−1^ represented amide B stretching. The characteristic PVA peaks at 1735 cm^−1^ and 848 cm^−1^ (carbonyl and CH_2_ stretching) were retained, along with DEW's. Amide I, amide II and amide III at 1651 cm^−1^, 1542 cm^−1^ and 1250 cm^−1^. The peak at 1078 cm^−1^ confirmed carbohydrate content in DEW. This result confirmed successful integration of DEW into the polymer matrix of PVA. The OH stretching absorption band, originally centred at 3062 cm^−1^ was observed to be broadened and shifted to 3035 cm^−1^, indicating successful incorporation of polar functional groups and enhanced intermolecular hydrogen bonding. Stronger hydrogen bonds cause OH absorption band to shift to lower wavenumbers within the 3650–3000 cm^−1^ region.^30,31^ The O–H stretching absorption in the PVA/Phage/DEW composite broadens to approximately 2000 cm^−1^, significantly beyond the conventional 3650–3000 cm^−1^ O–H stretching region. This unusually broad absorption arises from strong, cooperative hydrogen bonding networks formed between the hydroxyl groups of PVA and the amine and amide groups of DEW proteins and bacteriophage coat proteins. These O–H–N proton-sharing interactions involves partial hydrogen transfer between donor and acceptor, creating a broad and featureless absorption, continuing from ∼3500 cm^−1^ to ∼2000 cm^−1^. This phenomenon is well documented in PVA-protein and hydroxyl–amine composite systems.^32–34^ This is distinct from conventional O–H hydrogen bond shifting, which causes a moderate redshift within the 3650–3000 cm^−1^ range.

The observed redshift from 3062 cm^−1^ to 3035 cm^−1^ is therefore confirms increased polar oxygen groups on the surface. The absorption peaks corresponding to C–O and C–N stretching vibrations showed a distinct shift towards lower wave number from 1250 cm^−1^ and 1244 cm^−1^ and can be accounted for reactive oxygen species generated vacuum plasma treatment abstract hydrogen from alkyl groups, forming polar oxygen-containing functional groups.^35,36^ This incorporation is clearly evident from shift in absorption band corresponding to OH stretching (3062 cm^−1^ to 3035 cm^−1^), carbonyl (1735 cm^−1^ to 1730 cm^−1^), C=O (1651 cm^−1^ to 1641 cm^−1^), and C–O (1078 cm^−1^ to 1072 cm^−1^). These shifts confirm plasma treatment promotes hydrogen bonding networks though surface oxidation. Most chemical shifts and intensity changes occurred between the 1 and 3 minutes plasma treatment. Extending treatment to 10 minutes caused saturation, with key peaks (3035 cm^−1^ and 1641 cm^−1^) remaining constant, surface functional group saturation where additional groups can no longer be effectively incorporated. At 10 minutes, C–C and CH stretching shifted further to 834 cm^−1^ (from 848 cm^−1^), indicating prolonged exposure induces polymer backbone etching and chain scissoring. The 5 minutes treatment showed increased absorption intensity at 1730 cm^−1^ compared to the 1 and 3 minutes samples, attributed to additional carbonyl groups (C=O stretching of aldehydes or esters) formed from oxidative degradation of PVA side chains under prolonged reactive oxygen species exposure.^35,36^ Thus, ATR-FTIR analysis confirmed successful incorporation of oxygen-containing polar functional groups (–OH, –COOH, C=O) onto the nanofiber surface. Where optimal plasma treatment duration is 1–3 minutes maximizes functional group incorporation and hydrogen bonding while prolonged exposures (≥5 minutes) led to surface degradation.

### 4.3 Structural analyses

**Fig. 3:**
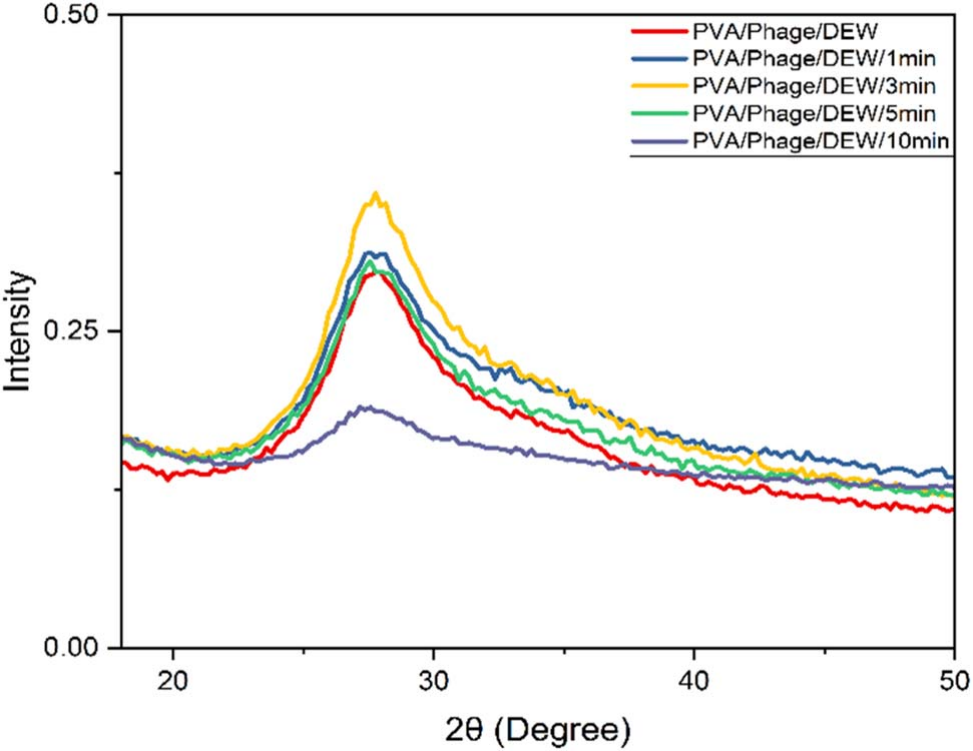
XRD analysis of untreated and plasma-treated PVA/Phage/DEW composites.

XRD analysis was performed to evaluate the crystalline properties of untreated and plasma-treated PVA/Phage/DEW nanofiber mats (Fig. 3). Crystallinity significantly influences mechanical and thermal properties. Higher crystallinity enhances hardness, stiffness, and melting point, but may also reduce the ductility and increasing brittleness.^37^

Untreated PVA/Phage/DEW nanofibers showed 30.36 ± 0.43% crystallinity while O_2_ plasma treatment for 1 and 3 minutes increased crystallinity to 38.64 ± 1.06% and 46.66 ± 1.10%, respectively, representing improvement of 27.3% and 53.7% over the untreated mats. This enhancement occurs because reactive plasma species selectively etch disordered amorphous regions, increasing the proportion of crystalline domains. The removal of amorphous material promotes molecular ordering, chain rearrangement, and cross-linking, collectively improving crystallinity.^37^ However, extended plasma treatment (5 and 10 minutes) decrease crystallinity to 32.80 ± 1.04% and 26.50 ± 1.08%, respectively. These improvements in crystallinity are observed after 1 and 3 minutes of plasma treatment, that can be attributed to etching of the amorphous regions resulting in increases in area of crystalline structures.^38^ This process promotes molecular ordering, facilitating cross-linking and chain rearrangement, thereby improving mechanical properties, such as tensile strength and hardness.^38,39^ However prolonged exposure (≥5 minutes) disrupts crystalline structures through excessive surface etching by reactive plasma species, introducing lattice defects amorphization.^40^ This reduced crystallinity correlates with diminished mechanical properties as lower crystallinity is associated with reduced strength and hardness. The optimal treatment window is 1–3 minutes, which maximizes crystallinity driven mechanical improvements without structural degradation. Beyond 3 minutes, prolonged exposure damages the crystalline architecture, negatively affecting overall performance. These findings demonstrate that controlled oxygen plasma treatment for 1–3 minutes provides an effective, non-destructive method for enhancing PVA/Phage/DEW nanofiber crystallinity and mechanical properties.

### 4.4 Mechanical properties

The mechanical properties of untreated and plasma treated PVA/Phage/DEW nanofibers were evaluated by measuring tensile strength and elongation-at-break (%), with results summarised in Table 4. The average thickness of the nanofibers remained relatively constant across all treatment durations, ranging from 0.220 ± 0.036 mm to 0.263 ± 0.021 mm (Table 4). This consistency in thickness confirms that the increase in tensile strength observed is a result of plasma-induced surface chemistry changes, rather than variations in the amount of material tested. The untreated PVA/Phage/DEW nanofibers exhibited a tensile strength of 3.86 ± 0.19 MPa and an elongation-at-break of 34.14 ± 1.50%. Upon exposure to oxygen (O_2_) plasma treatment, at 1 minute, tensile strength increased to 4.47 ± 0.49 MPa (a 15.80% improvement over the untreated nanofibers), while the elongation at break decreased to 29.59 ± 1.45%. At 3 minutes tensile strength reached 6.65 ± 0.55 MPa (72.28% improvement over the untreated mats and a 48.77% over 1 minute treatment), with elongation-at-break decreased further to 25.60 ± 1.94% (SI Fig. S2). These improvements are attributed to the plasma-induced incorporation of polar oxygenated functional groups on the nanofiber surface. The oxygenated species formed during O_2_ plasma treatment attaches to the nanofiber surface through hydrogen bonding and form hydrogen bonds among themselves, making a complex and reinforced surface structure, contributing to enhanced mechanical performance. This hydrogen-bonded assembly increases surface rigidity and restricts chain mobility, thereby stiffening the nanofiber mats, raising tensile strength, and reducing elongation at break.^41–43^

**Table 4:** Mechanical strength evaluation. Tensile strength (MPa), elongation at break (%), and nanofiber thickness for untreated and plasma-treated PVA/Phage/DEW nanofibers

Sample	Nanofiber thickness (mm)	Tensile strength (MPa)	Elongation at break (%)
PVA/Phage/DEW	0.258 ± 0.041	3.86 ± 0.2	34.14 ± 1.5
PVA/Phage/DEW/1 min	0.220 ± 0.036	4.47 ± 0.5	29.59 ± 1.4
PVA/Phage/DEW/3 min	0.263 ± 0.021	6.65 ± 0.6	25.6 ± 1.9
PVA/Phage/DEW/5 min	0.245 ± 0.023	3.29 ± 0.2	39.64 ± 1.8
PVA/Phage/DEW/10 min	0.253 ± 0.021	3.13 ± 0.4	46.23 ± 1.4

The observed mechanical trends across treatment durations correlate directly with the XRD crystallinity data as the 3 minutes sample, exhibited the highest crystallinity (46.66%), highest tensile strength (6.65 MPa) and lowest elongation at break (25.60%), consistent with the relationship between crystalline structure mechanical stiffness in semicrystalline polymers.^37–39^ Beyond 3 minutes, mechanical performance declined, as after 5 minutes, tensile strength decreased to 3.29 ± 0.21 MPa, while elongation at break rising to 39.64 ± 1.79%. At 10 minutes, tensile strength further declined to 3.13 ± 0.39 MPa with elongation-at-break increased to 46.23 ± 1.43%. This deterioration results from surface dehydration and etching effects that weakens polymer chains, consistent with reduced crystallinity (32.80% at 5 min and 26.50% at 10 min). The apparent increase in elongation at break at ≥5 minutes does not indicate improved ductility but rather reflects plasma induced chain scission, which reduces the average molecular weight of PVA chains and inter-chain entanglement, allowing greater extension (under comparatively low applied stress) before fracture, despite being overall weakening.^44^ Overall, a 3 minutes O_2_ plasma treatment provided the optimal surface activation and structural reinforcement for the PVA/Phage/DEW nanofibers, maximizing tensile strength while maintaining sufficient flexibility for wound dressing applications.

### 4.5 Contact angle and surface energy measurements

Contact angle measurement evaluates polymeric surface wettability. Although PVA being a water-soluble polymer, the use of CCD camera allowed to capture the initial water contact angle before significant penetration or dissolution of the nanofiber mat occurred. It was observed for the untreated nanofiber mats, the droplet was stable enough for measurement, where in case of plasma treated samples, the high surface energy led to more rapid spreading. Table 5 presents the water contact angle values for both the untreated and O_2_ plasma treated nanofibers PVA/Phage/DEW. Untreated PVA/Phage/DEW nanofiber exhibited an average water contact angle of 99.0 ± 5.9°. In contrast plasma-treated nanofibers showed significantly lower contact angles, highlighting enhanced hydrophilicity. After 1, 3 and 5 minutes plasma treatment, contact angles decreased to 70.8 ± 2.5°, 49.4 ± 6.6° and 38.6 ± 1.7° respectively, indicating significant improvements in the mat wettability. However, extending treatment from 5 to 10 minutes produced no further significant reduction in contact angle, suggesting surface saturation (provided in SI Fig. S3). Increased wettability is associated with increased surface energy on the polymer surface. Higher wettability can be associated with increased surface energy of a polymer surface, as O_2_ plasma treatment improves hydrophilicity by introducing oxygenated polar functional groups such as O, O_2_, O_2_^−^, COOH, C=O onto the surface.^45^ These polar functional groups interact strongly with water molecules, reducing the water contact angle and enhancing surface wettability.^46^ Plasma treatment significantly increases the polar components and overall surface energy (Table 5), confirming successful incorporation of polar functional groups onto the nanofiber surface. However, no notable changes in surface energy or its polar components were observed, when the treatment duration extended beyond 5 minutes, indicating surface reached saturation, where the density of functional groups could no longer be increased effectively. The optimal plasma treatment window is 1–3 minutes, which maximizes incorporation of polar group and surface wettability. This controlled surface activation enhances nanofiber performance for wound dressing applications by improving interaction with wound exudate and promoting cellular attachment.

**Table 5:** Contact angle and surface energy measurements of untreated and plasma-treated PVA/Phage/DEW nanofibers

Samples	Contact angle (°)		Polar (mJ m^−2^)	Dispersive (mJ m^−2^)	Total S.E. (mJ m^−2^)
	Water	Ethylene glycol			
PVA/Phage/DEW	99.0 ± 5.9	32.0 ± 0.4	1.6 ± 0.3	22.4 ± 1.6	24.0 ± 1.6
PVA/Phage/DEW/1 min	70.8 ± 2.5	30.2 ± 3.8	10.0 ± 2.3	31.4 ± 1.4	41.4 ± 2.7
PVA/Phage/DEW/3 min	49.4 ± 6.6	25.4 ± 0.2	35.3 ± 3.4	13.7 ± 2.3	49.0 ± 4.1
PVA/Phage/DEW/5 min	38.6 ± 1.7	25.2 ± 1.0	54.7 ± 3.5	6.9 ± 1.1	61.6 ± 3.6
PVA/Phage/DEW/10 min	35.4 ± 0.7	24.1 ± 0.8	55.4 ± 4.8	7.0 ± 0.8	62.4 ± 4.8

### 4.6 Water vapor transmission rate study

Maintaining appropriate water vapor transmission rate (WVTR) is crucial for effective wound healing. It has been reported that the cells in a desiccated wound may lose vitality and functionality, ultimately resulting in cell death.^47^ Additionally, dressings applied to dehydrated wound surfaces are prone to shrinkage, which may expose wound edges and compromise the bacterial barrier.^48,49^ So, maintaining an appropriate WVTR is therefore crucial to retain hydration and lock moisture in the wound site, thereby promoting faster healing.^47,49^ The ideal WVTR range for effective wound dressing is 2000–2500 g m^−2^ 24 h^−1^, with values closer to the lower limit (∼2000 g m^−2^ 24 h^−1^) being particularly suitable for practical clinical applications.^49^

**Fig. 4:**
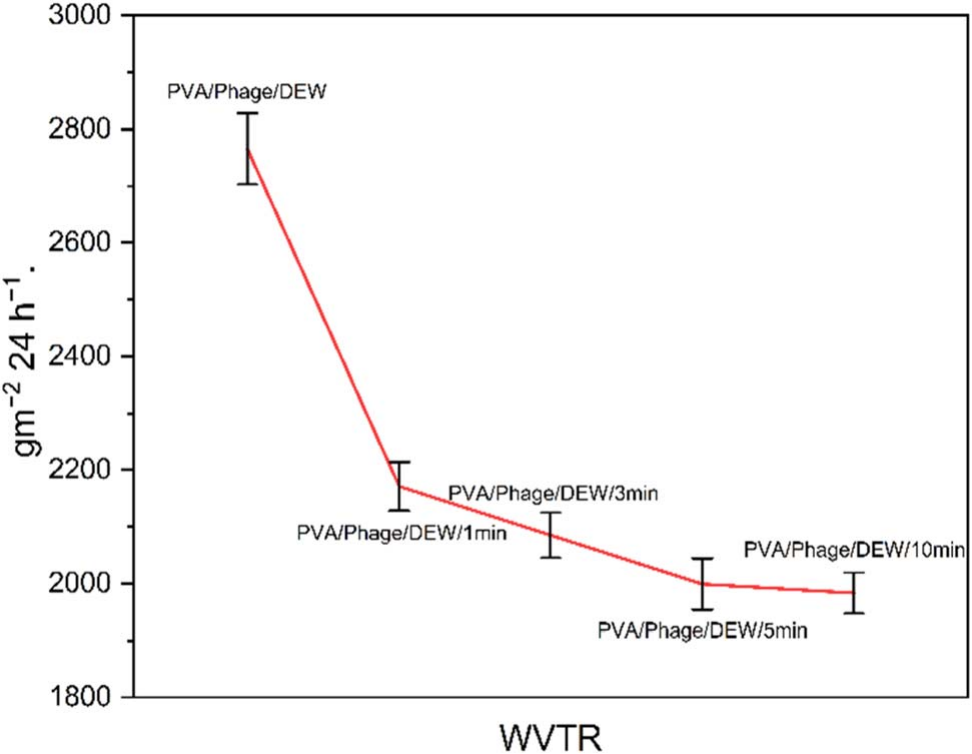
Water Vapor Transmission Rate (WVTR) analysis of untreated and plasma treated PVA/Phage/DEW nanofiber mats.

In Fig. 4, untreated PVA/Phage/DEW nanofiber mats exhibited WVTR of 2596 ± 49 g m^−2^ 24 h^−1^, exceeding the clinically recommended upper limit. Following O_2_ plasma treatment, WVTR values decreased progressively to 2117 ± 35 g m^−2^ 24 h^−1^, 2074 ± 29 g m^−2^ 24 h^−1^, 1981 ± 41 g m^−2^ 24 h^−1^ and 1973 ± 27 g m^−2^ 24 h^−1^ for treatment durations of 1, 3, 5 and 10 minutes, respectively. The reduction in WVTR is attributed to plasma-induced surface densification and cross-linking. O_2_ plasma introduces reactive oxygen species (ROS) that facilitate the formation of new intermolecular hydrogen bonds and covalent cross-links between PVA chains, creating a denser, more rigid surface layer. This increased molecular network density raises the tortuosity of water molecule diffusion pathways, reducing free volume available for water vapour transport. ATR-FTIR data confirmed the progressive strengthening of hydrogen bonding networks upon plasma treatment. XRD results demonstrated increased crystallinity at 1–3 min treatment durations (up to 46.66%), reflecting greater chain packing order and reduced water vapour permeability. The saturation of WVTR reduction observed beyond 5 minutes is consistent with the surface saturation of functional group incorporation observed in both the ATR-FTIR and contact angle data. The WVTR decreased consistently with increasing plasma treatment time, approaching saturation after 5 minutes, indicating that the plasma modified surface significantly enhances the nanofiber's ability to serve as a barrier to water vapour transmission. Similar plasma induced improvements in barrier properties have been previously reported, for polymeric films or scaffolds.^43^ However, the post-treatment WVTR values in this study remain close to the lower limits of the clinical range (∼2000 g m^−2^ 24 h^−1^), making these nanofibers favourable for maintaining a moist wound environment to support wound healing, as supported by prior studies.^49^

### 4.7 Antimicrobial test

**Fig. 5:**
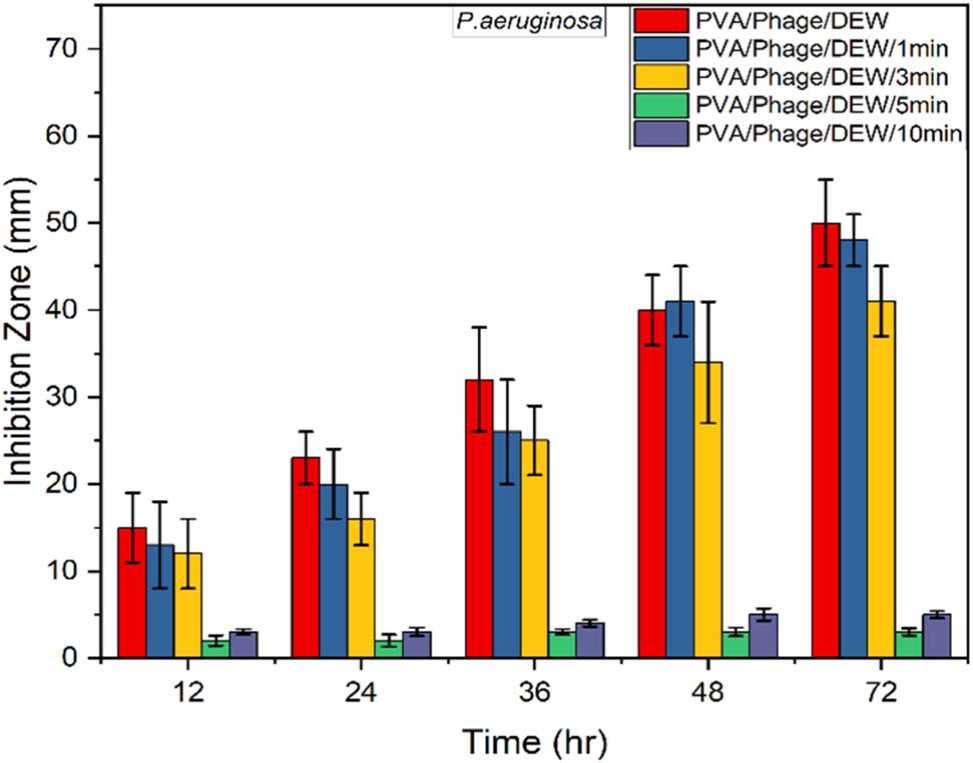
Zones of inhibition for untreated and O_2_ plasma-treated PVA/Phage/DEW nanofibers at varying treatment durations of 1, 3, 5, and 10 minutes against *Pseudomonas aeruginosa*.

Both untreated and plasma-treated PVA/Phage/DEW nanofibers exhibited antimicrobial activity against *P. aeruginosa* in agar plate. The untreated PVA/Phage/DEW nanofiber mats produced the maximum inhibition zone, with diameter of approximately 50 ± 5 mm after 72 h of incubation. In contrast, the O_2_ plasma treatment for 1 minute yielded a zone of 48 ± 3 mm, while the 3 minutes treatment decreased to 41 ± 4 mm. Noticeably, the PVA/Phage/DEW/5 min and PVA/Phage/DEW/10 min nanofiber mats exhibited no antibacterial activity (Fig. 5) Here, the zone of inhibition was considered as the primary metric for evaluating bioactivity because it provides a direct, semi-quantitative measure of the antimicrobial agent's ability to diffuse from the scaffold and effective neutralization of *P. aeruginosa*. This method is particularly informative for assessing whether plasma treatment duration preserves or destroys phage bioactivity and can be clinically relevant assay for evaluating the capacity of wound healing materials, to prevent microbial growth at the wound interface. However, plasma-treated nanofibers with longer treatment time exhibited smaller inhibition zone compared to untreated mats, with no inhibition zone at treatment times of 5 minutes or longer (provided in SI Fig. S4). This inverse relationship between plasma exposure time and antimicrobial efficacy is attributed to the biological inactivation of bacteriophages. It may be explained, as the structure of bacteriophage, which consists of a protein shell (capsid) that encloses its genetic material (DNA or RNA). As exposure to plasma increase with the treatment time, UV photons and reactive oxygen (ROS) generated during plasma^43^ can cause capsid disruption, thereby damaging bacteriophage's functionality that leads to loss of antibacterial activity.^50^ Hence, treatment duration of 5 minutes and above may have exceeded the threshold of the Phage on the nanofiber surface to be alive, thus showing ineffective against *P.aeruginosa.*

### 4.8 Haemolytic activity analysis

**Fig. 6:**
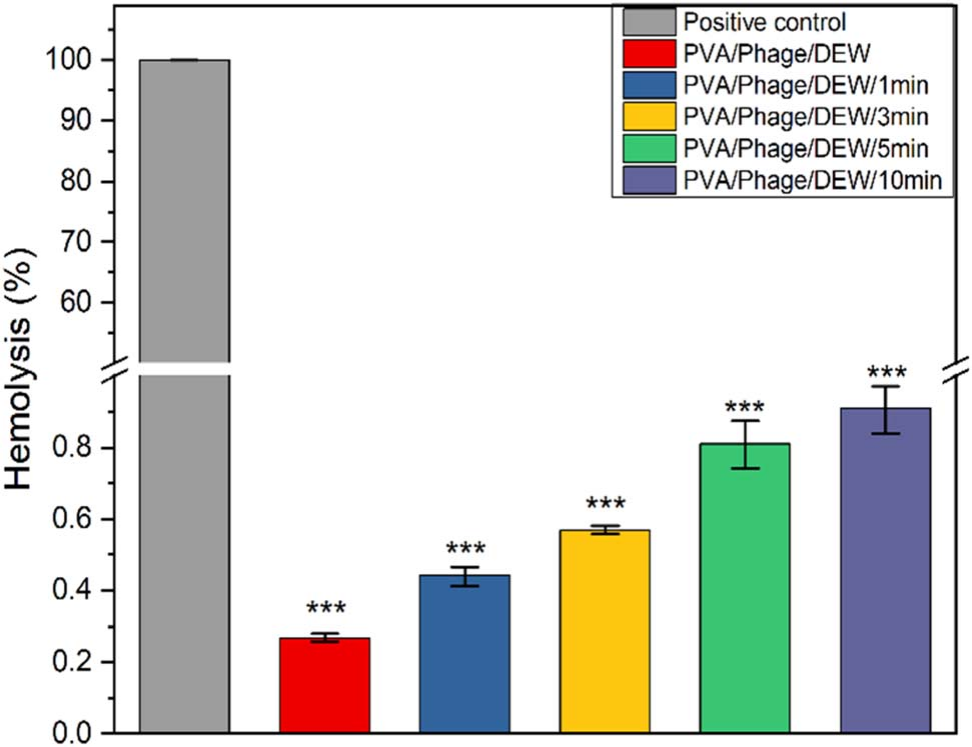
Hemocompatibility assessment of untreated and O_2_ plasma-treated (1–10 min) PVA/Phage/DEW nanofibers, compared to the positive control (Triton X-100).

For evaluating the potential of electrospun nanofibers as a wound dressing, their hemocompatibility was assessed using a haemolysis activity assay. In Fig. 6 both untreated and oxygen (O_2_) plasma-treated PVA/Phage/DEW nanofibers exhibited minimal haemolytic activity (<0.9%) suggesting excellent hemocompatibility compared to the positive control (100% cell lysis). Statistically there were no significant differences between the untreated and treated mats. Notably, the haemolysis activity of untreated nanofibers, and those treated for 1 and 3 minutes, was nearly similar. However, nanofibers subjected to prolonged plasma treatment (5 and 10 minutes) showed elevated haemolysis activity. These minor variations are discussed below by surface chemistry changes. While native egg whites do not show significant haemolytic activity, but some specific peptides or compounds derived may exhibit varying degree of haemolysis.^51^ Similarly, polyvinyl alcohol (PVA) generally exhibits low haemolytic activity.^52^ The O_2_ plasma act as a highly reactive etching agent due to the high electronegativity of oxygen. The ATR-FTIR confirmed successful incorporation of oxygen-containing polar functional groups, including hydroxyl (–OH), aldehyde (–CHO), carboxyl (–COOH), *etc.*, following O_2_ plasma treatment. This incorporation enhances surface hydrophilicity, as indicated by contact angle measurement, which makes it easier for interaction between the nanofibers and the cell surface that is covalent in nature, thereby contributing to minimal haemolysis.^53^ However prolonged plasma treatment (5 and 10 minutes) generates intermediate free radicals on the polymer surface.^54^ These free radicals may induce electrostatic interaction or oxidative stress upon contact with RBC cell membrane leading to destabilization of the lipid bilayer, thus contributing to a slight rise in haemolysis.^55^ Overall, the results indicate that O_2_ plasma treatment does not have adverse effect on the electrospun nanofibers. All samples-maintained haemolysis levels, well below the acceptable limit of 5%, confirming suitability for biological applications.

### 4.9 Antithrombogenic

The antithrombogenic properties of untreated and plasma-treated PVA/Phage/DEW nanofibers were evaluated, with results presented in Table 6. The data indicate that thrombus formation weight increases with prolonged blood coagulation time. The untreated PVA/Phage/DEW nanofibers exhibited thrombus formations that are comparable to the control, indicating that without plasma treatment, the fibres possess low antithrombogenic activity. Samples treated for 1 minute showed marginal improvement in antithrombogenic activity compared to the control. However, with further increase in treatment time of 3, 5 and 10 minutes, the PVA/Phage/DEW nanofibers begin to show a significant reduction in the weight of thrombus formation. The improvement in antithrombogenicity for treatment times of 3 minutes and above may be associated to more time for incorporation of polar functional groups onto the surface and thus enhanced surface free energies as observed by reduced contact angle. While the 1 minute treatment exhibited marginal improvement in antithrombogenic activity compared to that of the control. However, a distinct improvement in this property was observed with treatment time above 3 minutes. At observation period of 60 minutes, the 1 minute treated sample showed no significant change in antithrombogenicity, from the untreated samples, suggesting that under specific discharge condition, a short vacuum plasma treatment may not be effective in improving the blood compatibility of the nanofibers.

**Table 6:** Thrombus formation weight (mg) on control, untreated and plasma-treated PVA/Phage/DEW nanofibers after incubation periods of 15 and 60 minutes

Sample	Observation time	
	15 (min)	60 (min)
	Weight of thrombosis (mg)	
Control	49.3	82.4
PVA/Phage/DEW	47.6	78.1
PVA/Phage/DEW/1 min	42.2	74.5
PVA/Phage/DEW/3 min	38.8	66.0
PVA/Phage/DEW/5 min	35.1	59.2
PVA/Phage/DEW/10 min	32.9	56.4

### 4.10 Cytotoxicity

**Fig. 7:**
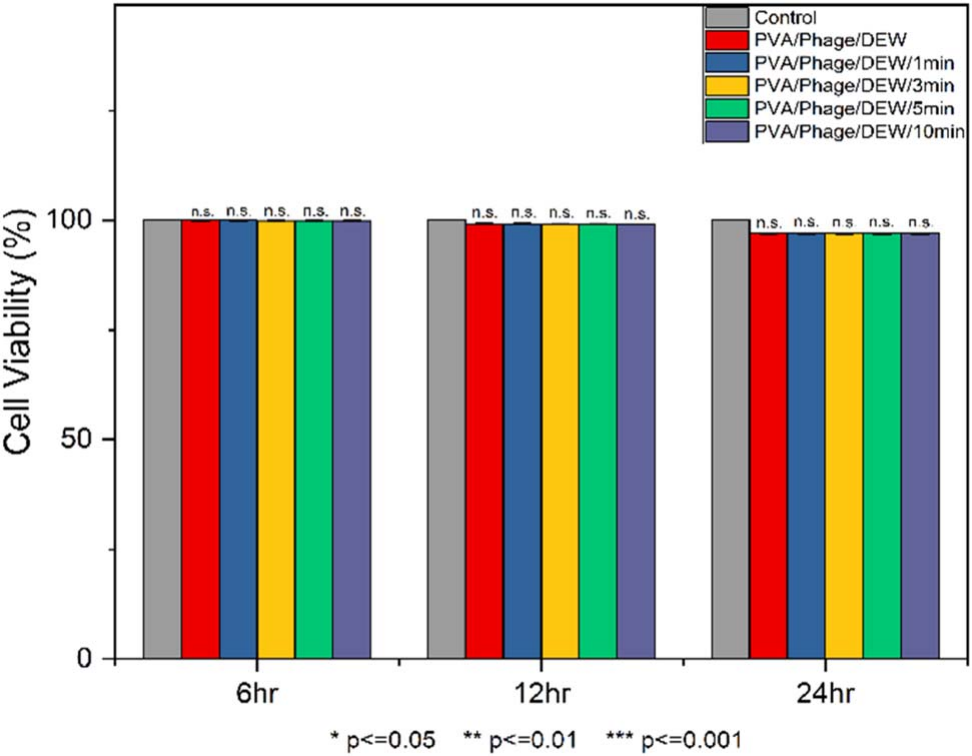
*In vitro* cytotoxicity evaluation. Cell viability (%) of RAW264.7 macrophage cells cultured with untreated and plasma-treated PVA/Phage/DEW nanofibers for 6, 12, and 24 hours. Data is expressed as mean ± SD (*n* = 3). The notation “n.s.” indicates non-significant differences compared to the control, confirming that the plasma-treated mats are non-cytotoxic.

The cytocompatibility of untreated and plasma treated PVA/Phage/DEW nanofibers mats were evaluated by measuring the viability of RAW264.7 macrophages after 6, 12, and 24 h of exposure to nanofibers mats, subjected to varying time period of plasma treatment (1 min, 3 min, 5 min, and 10 min) for 6, 12, and 24 h. After 6 h of incubation, cell viability was recorded as 99.95 ± 0.04% for untreated PVA/Phage/DEW, and 99.92 ± 0.03% for PVA/Phage/DEW/1 min, 99.88 ± 0.02% for PVA/Phage/DEW/3 min, 98.66 ± 0.17% for PVA/Phage/DEW/5 min, and 99.85 ± 0.02% for PVA/Phage/DEW/10 min, showing results comparable to those observed at 12 hours. At 24 h, the measured cell viabilities were 98.01 ± 0.04%, for untreated PVA/Phage/DEW nanofiber mats, 96.98 ± 0.03% for 1 min, 96.94 ± 0.02% for 3 min, 95.91 ± 0.04% for 5 min, and 95.90 ± 0.02% for 10 min. These findings indicate that oxygen plasma treatment does not induce any adverse effects on cell viability and the cytocompatibility of treated samples remains comparable to untreated and control samples across all tested time, up to 24 h. The results closely align with the haemolysis activity data, indicating that nanofibers treated with vacuum oxygen (O_2_) plasma for 1 and 3 min exhibit superior cell viability and hemocompatibility compared to those treated for longer time intervals (5 min and 10 min). This difference can be assigned to the generation of reactive oxygen species (ROS) or the formation of free radical on the polymer surface during prolonged plasma exposure.^56,57^ Overall, the studies confirm that controlled oxygen plasma treatment is safe and effective method for surface modification of PVA/Phage/DEW mats (Fig. 7).

## 5 Conclusions

This study demonstrates that controlled vacuum oxygen (O_2_) plasma treatment is a non-destructive method for surface modification of electrospun PVA/Duck Egg White (DEW) nanofiber mats incorporated with bacteriophage (vB_PaP_DMTU_1) for antibiotic-free wound dressing applications for combating multidrug-resistant *P. aeruginosa*. By systematically varying treatment duration from 1 to 10 minutes at constant power (144 W) and pressure (−100.3 kPa) we are able to alter the physicochemical, mechanical, antibacterial, and biocompatibility properties of the mats. A treatment duration of 1–3 minutes was identified as the optimal time window for surface modification while reserving bacteriophage viability. Within this window, O_2_ plasma selectively etched amorphous polymer domains, improving crystallinity from 30.36% (untreated) to a peak of 46.66% at 3 minutes. This structural ordering, combined with the incorporation of polar oxygen-containing groups (–OH, –COOH, C=O) confirmed by ATR-FTIR, produced a 72% improvement in tensile strength (6.65 ± 0.55 MPa at 3 min) and reduced water contact angle from 99.0° to 49.4, with an increase in total surface energy and wettability. WVTR values in the 1–3 minutes range (2117–2074 g m^−2^ 24 h^−1^) remained within the clinically recommended range for moist wound healing environment (2000–2500 g m^−2^ 24 h^−1^). Plasma treatment for 1–3 minutes fully preserved bacteriophage functionality yielding inhibition zones of 41–50 mm against multidrug resistant *P.aeruginosa*, confirming that short duration vacuum plasma is compatible with phage viability. Additionally, *in vitro* biocompatibility assessments validated the clinical potential of the optimized scaffolds. The nanofibers exhibited negligible haemolytic activity (<0.9%) and excellent cytocompatibility with RAW264.7 macrophages (>96% viability even after 24 h), with 1–3 minutes plasma treated samples exhibiting the highest cell viability among all group tested.

Beyond 3 minutes of plasma exposure, a decline in crystallinity and tensile strength was observed. This degradation, alongside a complete loss of antibacterial activity by 5 minutes, is attributed to the generation of UV photons and reactive oxygen species, which deactivate the phage *via* capsid disruption. While these findings are promising, certain limitations of the study should be acknowledged. EDX, used for elemental confirmation, has limited sensitivity for surface-confined functional groups thus XPS characterisation should be performed in future work to provide definitive chemical-state identification. In conclusion, these findings demonstrate that controlled vacuum O_2_ plasma treatment for 1–3 minutes transforms PVA/Phage/DEW nanofibers into a mechanically robust, phage-active, and highly biocompatible wound dressing platform capable of rapid, localized delivery of viable bacteriophages. This antibiotic-free surface engineering strategy provides an alternative approach for the targeted treatment of biofilm-forming, multidrug-resistant *P. aeruginosa* in chronic and burn wound care.

## Author contributions

Kaushik Kokil Nath: conceptualization, methodology, investigation, writing; original draft. Dhirangkana Bora: investigation, methodology (bacteriophage preparation, antimicrobial assays). Orison Waikhom: investigation (plasma treatment). Akuleti Saikumar: investigation (mechanical testing). Subrata Mishra: investigation (cytotoxicity evaluation). Bikash K. Das: investigation (contact angle analysis). Bibhusita Baishya: data curation. Biplob Mondal: supervision, resources. Laxmikant S. Badwaik: supervision, resources. Nirmal Mazumder: supervision, review & editing. Manabendra Mandal: resources. Suman Dasgupta: resources. Rajib Biswas: supervision, review & editing. Gazi Ameen Ahmed: supervision, project administration, review & editing.

## Conflicts of interest

There are no conflicts to declare.

## Supplementary Material

RA-016-D6RA01532H-s001

## Data Availability

The authors declare that the data supporting the findings of this study are available from the corresponding authors upon reasonable request. Supplementary information (SI): SEM images of PVA/phage/DEW nanofibers at varying PVA:DEW ratios (Fig. S1); mechanical properties including tensile strength, elongation at break, and Young's modulus of untreated and plasma-treated nanofibers (Fig. S2); contact angle analysis demonstrating wettability variation across plasma treatment durations (Fig. S3); antibacterial inhibition zone images against *Pseudomonas aeruginosa* at multiple time points (Fig. S4); and quantitative inhibition zone measurements (mm) over a 72-hour period for nanofibers treated with varying plasma durations (Table S1). See DOI: https://doi.org/10.1039/d6ra01532h.
