# Supplementary material for: Effect of vacuum plasma treatment duration on physicochemical, mechanical, and biocompatibility properties of bacteriophage-incorporated PVA/duck egg white nanofibers
Source: RSC Adv. 2026 Jul 2;16(34):32202–16. doi: 10.1039/d6ra01532h (PMC13325934; doi:10.1039/d6ra01532h)
Supplement: RA-016-D6RA01532H-s001 [file RA-016-D6RA01532H-s001.pdf]

Supplementary Information (SI) for RSC Advances.
This journal is © The Royal Society of Chemistry 2026

Supplementary Materials

Effect of Vacuum Plasma Treatment Duration on Physicochemical, Mechanical, and Biocompatibility Properties of Bacteriophage-Incorporated PVA/Duck Egg White Nanofibers

Kaushik Kokil Nath[1], Dhirangkana Bora[2], Orison Waikhom[3], Akuleti Saikumar[4], Subrata Mishra[5], Bikash K Das[6], Bibhusita Baishya[1], Biplob Mondal[3], Laxmikant S. Badwaik[5], Nirmal Mazumder[7], Manabendra Mandal[2], Suman Dasgupta[4], Rajib Biswas[1*], Gazi Ameen Ahmed[1]

[1] Laboratory for Plasma Processing of Materials, Department of Physics, Tezpur University, Tezpur, 784028, Assam, India,

[2]Applied Microbiology and Biotechnology Laboratory, Department of Molecular Biology and Biotechnology, Tezpur University, Tezpur, India,

[3]Sensors and System Engineering Laboratory, Department of Electronics and Communication Engineering, Tezpur University, Tezpur, India,

[4]Department of Food Engineering and Technology, Tezpur University, Tezpur, India

[5] Metabolic Disease Biology Laboratory, Department of Molecular Biology and Biotechnology, Tezpur University, Tezpur, India,

[6]Nanoscience and Soft-Matter Laboratory, Department of Physics, Tezpur University,

[7]Manipal Academy of Higher Education, Manipal campus, India.

Corresponding Author: nirmal.mazumder@manipal.edu

Supplementary Figure S1: SEM Images for Varying PVA: DEW Ratios

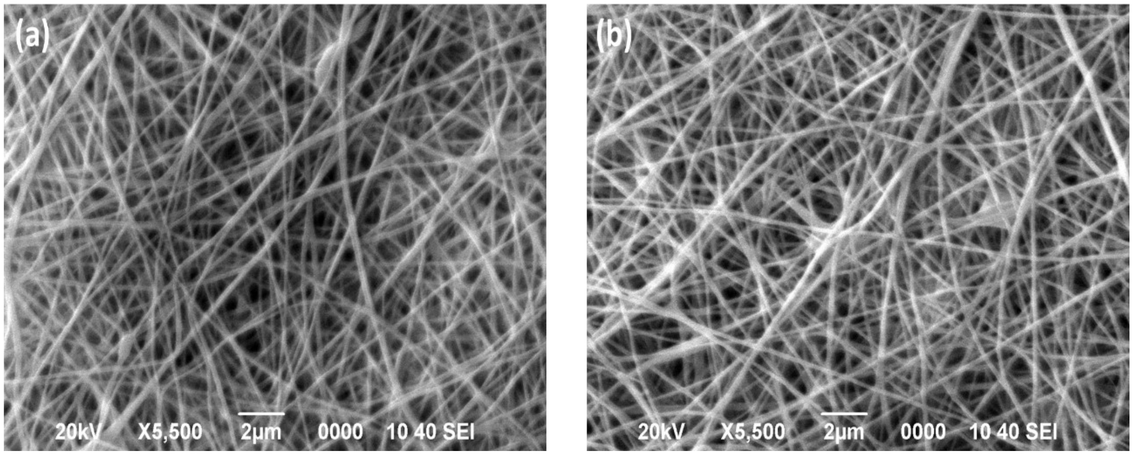

Figure S1: (a) PVA/Phage/DEW nanofibers (PVA: DEW 1:1) and (b PVA/Phage/DEW nanofibers (PVA: DEW 1:2) at 5,000× magnification showing bead formation due to increased viscosity.

Supplementary Figure S2: Mechanical Strength Analysis

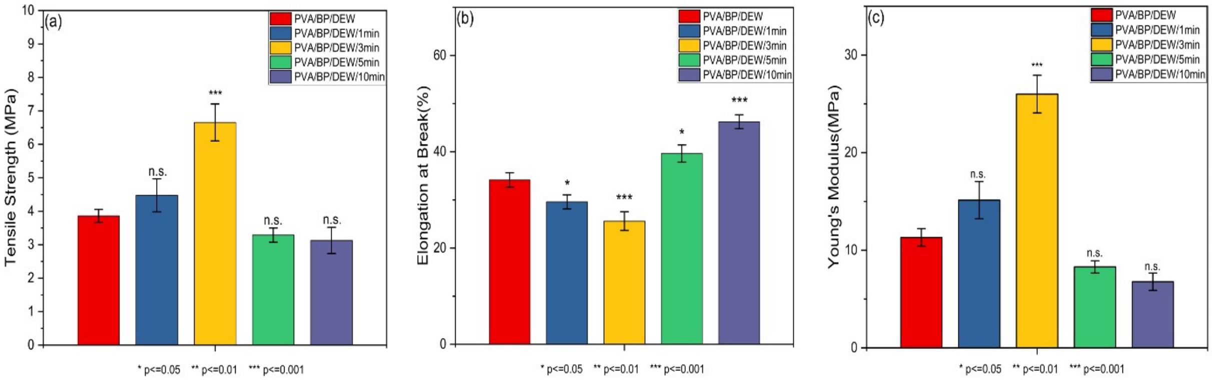

Figure S2: Mechanical properties of PVA/Phage/DEW nanofibers. (a) Tensile strength, (b) elongation at break, and (c) Young's modulus of untreated and plasma-treated nanofibers (1, 3, 5, and 10 minutes). Significance levels are denoted as * $p \leq 0.05$, ** $p \leq 0.01$, and *** $p \leq 0.001$; n.s. indicates non-significant differences.

Supplementary Figure S3: Contact Angle analysis

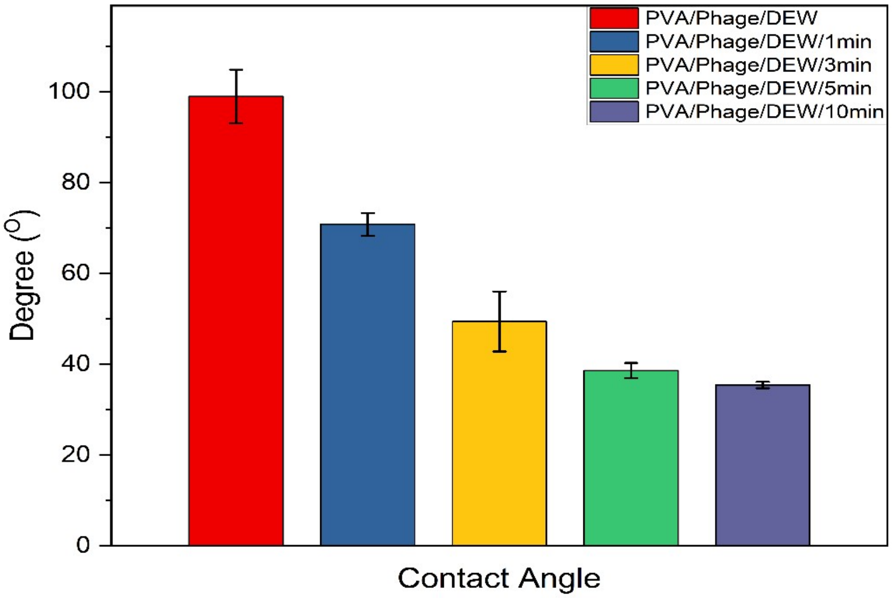

Figure S3: Contact angle analysis of PVA/Phage/DEW nanofibers, demonstrating variation in wettability for nanofibers untreated and exposed to O_2 plasma for durations of 1, 3, 5, and 10 minutes.

Supplementary Figure S4: Antibacterial Activity Against *Pseudomonas aeruginosa*

Incubation Time	NP	1 min	3 min	5 min	10 min
12 hr	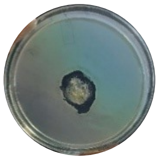	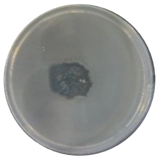	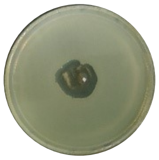	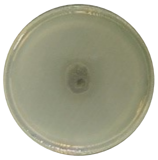	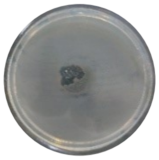
24 hr	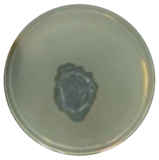	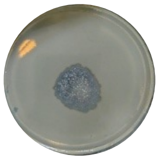	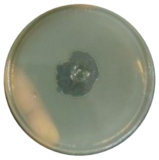	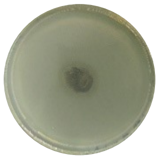	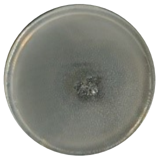
36 hr	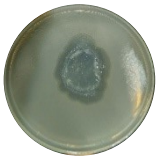	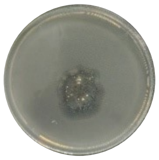	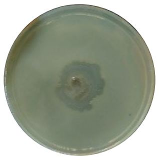	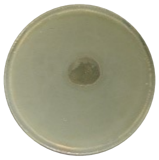	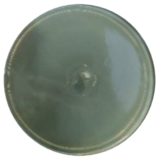
48 hr	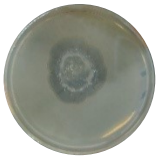	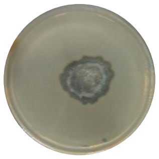	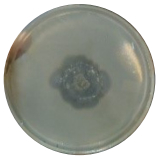	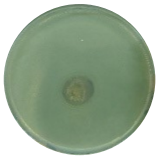	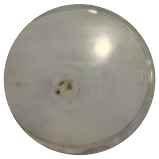
72 hr	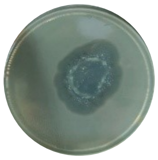	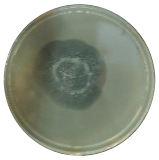	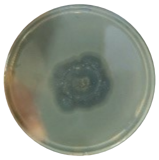	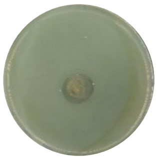	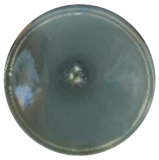

Figure S4: Images of inhibition zones for untreated and plasma-treated PVA/Phage/DEW nanofibers at different time interval; (1, 3, 5 and 10min) against *Pseudomonas aeruginosa* at 12, 24, 36, 48 and 72 hours.

Supplementary Table S1: Antibacterial inhibition zone measurements (mm) against *Pseudomonas aeruginosa* for PVA/Phage/DEW nanofibers treated with varying plasma durations over a 72-hour period.

Pseudomonas aeruginosa					
**Time (hr)**	**Non-Plasma**	**1min**	**3 min**	**5 min**	**10 min**
**12**	15 ± 4	13 ± 5	12 ± 4	2 ± 0.6	1 ± 0.3
**24**	23 ± 3	20 ± 4	16 ± 3	2 ± 0.7	1 ± 0.5
**36**	32 ± 6	26 ± 6	25 ± 4	3 ± 0.3	2 ± 0.4
**48**	40 ± 4	41 ± 4	34 ± 7	3 ± 0.5	3 ± 0.7
**72**	50 ± 5	48 ± 3	41 ± 4	3 ± 0.4	3 ± 0.4
